# Managing multimorbidity in primary care in patients with chronic respiratory conditions

**DOI:** 10.1038/npjpcrm.2016.43

**Published:** 2016-09-15

**Authors:** Deborah Morrison, Karolina Agur, Stewart Mercer, Andreia Eiras, Juan I González-Montalvo, Kevin Gruffydd-Jones

**Affiliations:** 1 General Practice and Primary Care, Institute of Health and Wellbeing, University of Glasgow, Glasgow, UK; 2 Life and Health Sciences Research Institute (ICVS), School of Health Sciences, University of Minho, Braga, Portugal; 3 ICVS/3B's-PT Government Associate Laboratory, Braga/Guimarães, Portugal; 4 Rainha D. Amélia Family Health Unit, Porto, Portugal; 5 Geriatrics Department, IdiPaz Research Institute Hospital Universitario La Paz, Universidad Autónoma de Madrid, School of Medicine, Madrid, Spain; 6 Box Surgery, Wiltshire, UK

## Abstract

The term multimorbidity is usually defined as the coexistence of two or more chronic conditions within an individual, whereas the term comorbidity traditionally describes patients with an index condition and one or more additional conditions. Multimorbidity of chronic conditions markedly worsens outcomes in patients, increases treatment burden and increases health service costs. Although patients with chronic respiratory disease often have physical comorbidities, they also commonly experience psychological problems such as depression and anxiety. Multimorbidity is associated with increased health-care utilisation and specifically with an increased number of prescription drugs in individuals with multiple chronic conditions such as chronic obstructive pulmonary disease. This *npj Primary Care Respiratory Medicine* Education Section case study involves a patient in a primary care consultation presenting several common diseases prevalent in people of this age. The patient takes nine different drugs at this moment, one or more pills for each condition, which amounts to polypharmacy. The problems related with polypharmacy recommend that a routine medication review by primary care physicians be performed to reduce the risk of adverse effects of polypharmacy among those with multiple chronic conditions. The primary care physician has the challenging role of integrating all of the clinical problems affecting the patient and reviewing all medicaments (including over-the-counter medications) taken by the patient at any point in time, and has the has the key to prevent the unwanted consequences of polypharmacy. Multimorbid chronic disease management can be achieved with the use of care planning, unified disease templates, use of information technology with appointment reminders and with the help of the wider primary care and community teams.

## Case study

Mr A is a 65-year-old unemployed man who lost his job a year ago, having been a factory worker for many years. He is married and lives in subsidised public housing. His wife was diagnosed with breast cancer 9 months ago, and underwent a mastectomy and chemotherapy but is now doing well. They have two grown-up children, both living overseas. He was recently diagnosed with chronic obstructive pulmonary disease (COPD).

His main complaint at these recent consultations has been a productive cough and shortness of breath especially at night. His sleep has been poor, which he relates to his cough. Sometimes he takes medication to be able to have a full night’s sleep. He is also known to have hypertension, and general osteoarthritis affecting several joints and the back. He had a myocardial infarction 18 months ago, and was also found to have mild heart failure (left ventricular systolic dysfunction). He is on numerous medications, including inhalers and tablets (see the table below). He continues to smoke >20 cigarettes per day and admits to drinking more alcohol recently, as he feels it helps him sleep.

Sometime after his wife was diagnosed with cancer, shortly after his myocardial infarction, he was prescribed anti-depressive medication, which he takes irregularly, as well as a tablet to help him sleep.

He has different annual reviews by different practices nurses for his different long-term conditions. He often does not turn up for these booked reviews.[Table tbl5]

## The epidemiology of multimorbidity in chronic respiratory conditions

This case involves a patient in a primary care consultation presenting with at least six common diseases prevalent in people of this age.

## Multimorbidity

The term multimorbidity is usually defined as the coexistence of two or more chronic conditions within an individual, whereas the term comorbidity has traditionally been used to describe patients with an index condition (such as asthma) and one or more additional conditions.^1^ Multimorbidity is becoming the preferred terminology in primary care, where the definition of an index condition is less meaningful. The prevalence of multimorbidity has doubled over the last 20 years,^2^ and in many countries is now the norm rather than the exception in the elderly and in patients with chronic disease.^3,4^ Multimorbidity is associated with lower quality of life, lower physical function, higher mortality rates,^5^ and also increased polypharmacy and higher acute admission rates,^6^ all of which lead to increased burdens for patients and contribute to increased costs for health-care providers. There is a lack of consistency in the prevalence rates described in the literature. This can be partially explained by variable disease reporting methods, the number of conditions ‘included’ and the type of populations studied. However, studies consistently show that multimorbidity increases with age.^3,4^ However, this does not mean that multimorbidity is only an older person’s problem. In many countries there are more people under the age of 65 years living with multimorbidity than over.^3,4,7^

## Multimorbidity and respiratory conditions

### Asthma

Respiratory conditions are among the most common chronic conditions in patients with multimorbidity. In a nationally representative primary care study of 1.75 million people in Scotland, currently treated asthma had a prevalence of 6% and (despite the fact that asthma affects many children) 52% of individuals with asthma had one or more additional conditions.^3^ The most common comorbidities in this Scottish study are shown in Table 1. Similar findings have been reported in other population-based surveys.^8,9^ For example, Steppuhn *et al*.^9^ recently conducted a national survey of 43,312 people in Germany in which 1,134 individuals reported asthma (5%) and found that 58% of those with asthma had at least one comorbidity. Having three or more chronic conditions was more than twice as common in those with asthma (17%) compared with those without asthma (8%). Zhang *et al*.^8^ conducted a survey of 132,221 Canadians aged 12 years and over, of whom 10,089 (8%) had asthma, and reported that 59% of those with asthma had at least one other chronic illness. After adjusting for age and gender, all major chronic conditions except cancer were more common in those with asthma than in those without, similar to findings reported by Steppuhn *et al*.

As with other studies,^10^ Steppuhn *et al.* also comment that comorbidities in asthma are not limited to the elderly, showing that over 30% of those aged 18–44 with asthma have at least one other chronic condition.^9^ This raises concerns that these younger patients may be on a trajectory towards higher burdens of multimorbidity as they age.^10^ Comorbidity in asthma appears to lead to worse outcomes, with higher risk for exacerbations,^8^ unscheduled care^9^ and mortality.^11^

### Chronic obstructive pulmonary disease

In the study by Barnett *et al.*,^3^ COPD had a prevalence of 3%, and 82% of these patients had two or more additional conditions (47% had three or more); however, there are wide variations in the prevalence rates of COPD, never mind multimorbidity prevalence rates, making it impossible to confidently provide a true figure.^12,13^ The most common comorbidities in the Scottish study are shown in Table 1. A study in Ireland found that 60% of patients with chronic respiratory disease had at least one or more other chronic conditions, rising to 90% among individuals over 70 years of age.^14^ COPD is predicted to be the third leading cause of death globally by 2020,^15^ and mortality rates in chronic respiratory conditions are substantially increased by comorbidities.^16^ The number of comorbidities in patients with COPD is also related to worse symptom control and more exacerbations.^17^ The same study also showed that the type of morbidities commonly associated with COPD were often life-threatening conditions such as ischaemic heart disease (present in almost 24% of COPD patients).

COPD is also associated with polypharmacy and this will be substantially increased in those with comorbidities, thus increasing the risk for adverse drug reactions, drug–drug interactions and drug–disease interactions. This can increase the treatment burden as well as lower medication adherence.^18^ In addition, health professionals may under-prescribe for one condition (e.g., beta-blockers for cardiac disease) for fear of causing worsening respiratory symptoms when both conditions are present, leading to patients missing out on potentially beneficial medications, as seen in the case study where his bisoprolol dose is likely to be suboptimal.

## Respiratory disease and comorbid mental health conditions

Although patients with chronic respiratory disease often have physical comorbidities, they also commonly experience psychological problems such as depression and anxiety. It has been reported that up to 25% of people with COPD^19,20^ and 13–14% of people with asthma^9,21^ have depression, whereas only 7% of people without these chronic respiratory conditions have depression. The presence of mental health conditions negatively influences the outcome for chronic conditions including exacerbations and acute admissions.^6,9,21–25^

It is interesting to note that depression is among the top five comorbidities in both asthma and COPD, along with chronic pain (Table 1).

The relationship between chronic respiratory disease (and indeed most chronic diseases) and mental health problems is likely to be bidirectional; patients with chronic respiratory conditions may develop depression and anxiety as a result of their respiratory conditions, perhaps due to functional limitations, inability to work, etc. Patients with mental health conditions are at a high risk of developing chronic physical conditions, because of lifestyle factors such as higher rates of smoking. Smith *et al*.,^26^ for example, reported that patients with depression have a 70–80% increased risk of having asthma or COPD.

## Gender

Previous studies have shown that multimorbidity is generally more prevalent in women than in men.^4^ Asthma tends to be more prevalent in women than in men,^27^ whereas historically COPD tended to have the opposite gender balance,^28^ although there is evidence that this trend is starting to reverse.^29,30^ To date, however, there is no clear evidence of a difference in the rates of comorbidity by gender, specifically in those with asthma.^8,9^ This is in contrast to COPD, in which gender differences in comorbidity have been reported,^31^ finding a mean number of comorbidities of 4.6 (s.d. 3.2) for females and 6.2 (s.d. 3.5) for males (*P*<0.001).

## Multimorbidity and socioeconomic status

The prevalence of multimorbidity is socially patterned, being higher in people of lower socioeconomic status.^4^ People living in areas of high socioeconomic deprivation develop multimorbidity 10–15 years earlier than those living in affluent areas.^3^ This relationship between deprivation and multimorbidity is particularly visible in patients with a combination of mental and physical health problems, with a two- to threefold greater prevalence of mental–physical multimorbidity in deprived areas compared with affluent areas.^3,10^ This is reflected in respiratory diseases; for example, 23% of patients with COPD living in deprived areas have diagnosed depression, compared with 14% of COPD patients in affluent areas.^3^ Again a similar picture is seen with asthma, where lower levels of educational attainment are associated with comparatively high rates of 3+ comorbidities.^9^ A further study using administrative data from 34,741 Canadian patients aged 18–70 years with obstructive respiratory disease (asthma and COPD combined) demonstrated a relationship between socioeconomic status (defined by income quintile) and comorbidity, with lower socioeconomic status individuals having three times the levels of high comorbidity (⩾4) compared with those within the higher socioeconomic groups (7.4% vs. 2.5%, respectively).^32^

## Future challenges

Multimorbidity of chronic conditions, including respiratory conditions, markedly worsens outcomes for the patients, increases treatment burden and increases health service costs.^33^ There are very few interventions in primary care specifically developed for patients with multimorbidity in general or with respiratory disease in particular. Indeed, many trials exclude such patients. Thus, guidelines are based on the evidence that may or may not be of relevance in patients with respiratory conditions, and recommendations rarely take account of multimorbidity. The importance of the generalist clinician, who can deal effectively with complexity and manage not just respiratory conditions but also the associated physical, mental and social comorbidities, cannot be over-stated. This is especially true in areas of high deprivation, given the social patterning of multimorbidity and its burden.

Epidemiological studies of multimorbid patients, especially those of a longitudinal nature, may help researchers identify target groups and fruitful areas for intervention. Interventions are likely to be complex and will require appropriate methodologies to evaluate them. Increasingly, this will probably require mixed-methods approaches and cost-effectiveness evaluation.

## Multimorbidity and polypharmacy

Multimorbidity is associated with health-care utilisation and specifically with an increased number of prescription drugs in individuals with multiple chronic conditions such as COPD.^34^ Mr. A takes nine different drugs at this moment, one or more pills for each condition, which amounts to polypharmacy.

There is no consensus in defining polypharmacy, but commonly used definitions are ‘>5’ or >10 medications taken regularly by an individual, and is a common situation among older people.^35^

In clinical practice, polypharmacy is the rule in patients with cardiac and respiratory diseases. Patients with cardiac conditions such as heart failure and coronary artery disease currently receive multiple drugs simultaneously for controlling both the main condition and risk factors. In respiratory diseases such as COPD several drugs are frequently needed to improve control of the symptoms, and polypharmacy is commonly used, which substantially contributes to direct medical costs in COPD.^36^ Overuse of respiratory medication is an important risk factor for adverse effects.^37^ Polypharmacy and the prescription of multiple daily doses contribute to poor patient adherence in COPD, which is a major concern.^38^

Although polypharmacy with respiratory drugs is an indicator of poor health status in COPD, a direct negative effect of multiple medications on health status cannot be excluded. Polypharmacy is associated with poor self-rated health status among all age groups in the general population.^37^

Older people are the main consumers of drugs due to the increasing incidence of chronic disease with advancing age.^39^ Pharmacotherapy in the elderly requires knowledge of the age-dependent changes in pharmacokinetics and pharmacodynamics, as well as an assessment of comorbidity and concurrent drug therapy to reduce adverse effects.

The factors predisposing to polypharmacy are poor health, multiple chronic diseases, multiple prescribing physicians, the patient’s expectations (e.g., in relation to therapeutic advances) and education, an increasing demand for health care, supplemental insurance, and reluctance to discontinue old medications. Polypharmacy increases the risk for inappropriate medications,^40^ nonadherence to treatments, morbidity, mortality and adverse drug reactions.^37^

Depending on the circumstances (e.g., why and how drugs are being administered), polypharmacy can be appropriate (potential benefits outweigh potential harms) or inappropriate (potential harms outweigh potential benefits).^35^

## Potentially inappropriate medication: current tools

Inappropriate prescribing in the elderly population is an emerging health issue and comprises the following: use of an inappropriate dose, formulation, duration and delivery of drugs; use of unnecessary drugs; omission of necessary medicines; and the risk of drug interactions and adverse drug events.^39^ Errors in prescribing and in the omission of medicines are highly prevalent among medically stable older people in primary care as well.^41^

Inappropriate medications may be detected with screening tools for the assessment of the quality and safety of prescriptions. The Beers criteria^42^ constitute one of the most widely used tools for assessing the inappropriateness of prescriptions. They were published in 1991, revised in 1997, and updated in 2002 and 2012. The 2002 updated version was designed for all older people and not just for nursing home residents (as were the two previous versions).^43^ An alternative is the STOPP (Screening Tool of Older Persons’ Prescriptions) and START (Screening Tool to Alert doctors to the Right Treatment) criteria.^44^

Respiratory pharmacology is under-represented in the 2012 Beers criteria and, in the 2002 version of the list, propranolol and long-acting benzodiazepines (chlordiazepoxide, chlordiazepoxide-amitriptyline, clidinium-chlordiazepoxide, diazepam, quazepam, halazepam and chlorazepate) are identified as inappropriate prescriptions for older adults with COPD, although benzodiazepines may be used to palliate intractable breathlessness. They do not sufficiently address other drug–disease interactions, drug misuse and under- or overprescribing.

Mr. A takes zolpidem irregularly; in the Beers criteria, although comparable to benzodiazepine, it is included in the non-benzodiazepine hypnotics group, which is not considered inappropriate medication specifically in the context of COPD disease. It is considered inappropriate medication if taken for >90 days, independently of the clinical presence of COPD.

The STOPP and START criteria are able to detect inappropriate medications (drug class duplication and drug–drug and drug–disease interactions) and the omission (or underprescription) of indicated drugs, respectively. They have shown better sensitivity than the Beers criteria in identifying prescription problems and in identifying patients who required hospitalisation as a result of inappropriate prescription-related adverse events.^45^ The START criteria can help clinicians consider the benefit (or otherwise) of starting new drugs in complex clinical situations. The main principles in correctly prescribing for COPD with STOPP/START criteria are summarised in Table 2.

Mr. A already takes an inhaled β_2_-agonist and anticholinergic agent according to the START list (Table 3). Use of these screening tools can help prevent inappropriate prescriptions and improve appropriate prescribing with a reduction in adverse drug events, costs, nonadherence and polypharmacy.

## Compliance

Polypharmacy hinders compliance with drug treatment. Patient adherence to prescribed drugs has been shown to be low, especially in long-term treatment.^46^ Nonadherence to treatments can be influenced by several factors such as the complexity of the dosing schedule, frequent changes in medication, multiple medication, side effects, the cost of drugs, difficult routes of administration, difficult-to-open containers, cognitive impairment, visual impairment, inadequate patient education or understanding, and the impairment of physical function.^47^

Inhalation is the preferred mode of delivery for many drugs in the treatment of airway diseases such as COPD. The advantage of this route is the delivery of drugs to the site where they are needed. As a result, it allows smaller doses to be administered, which are effective with a much lower risk for side effects,^48^ which may improve patient compliance.

Inhaled drugs can be delivered by nebulisers, pressurized metered-dose inhalers (pMDI), dry-powder inhalers (DPI) and soft mist inhalers. Moreover, therapeutic efficacy depends on adequate airway drug deposition, which is influenced by particle size and inhalation techniques. A pMDI requires good actuation/inhalation coordination for optimal lung deposition, often difficult for elderly patients. The soft mist inhaler requires a slow, deep breath, whereas a DPI requires sufficient inspiratory flow.

The inability to produce an adequate peak inspiratory flow is critically important. Both physiological age-related changes and airway diseases can lead to weak inspiratory manoeuvres. The age-related reduction in thoracic compliance and in diaphragmatic strength associated with the COPD-related decline in inspiratory muscle function due to lung hyperinflation can impair the generation of an adequate peak inspiratory flow. Further difficulties with the delivery devices may arise because of visual limitations, cognitive deterioration, poor coordination and arthritis, which impair device handling, which could be a hindrance to optimum disease control in patients with multimorbidity.^49^

Mr. A smokes and takes both a short-acting β_2_-agonist and a long-acting anticholinergic for the treatment of his COPD. If more than one drug is indicated, the use of combination inhalers of long-acting β_2_-agonist and long-acting anticholinergic or long-acting β_2_-agonist and inhaled steroids increases adherence to the treatment.

Re-evaluate the COPD category, and, if clinically appropriate and necessary, changing to a combination of long-acting β_2_-agonist/long-acting anticholinergic in a single inhaler might be more effective at producing maintained symptom relief than short-acting bronchodilators, and the convenience might improve compliance.

## Adverse drug reactions

In the elderly, the use of numerous medications associated with pharmacokinetic and pharmacodynamic changes can lead to adverse drug reactions, defined as any injury resulting from drug therapy.^49^ These may be drug–drug interactions, drug–disease interactions, drug–food interactions or drug–herb interactions.^39^

The risk of these events is exacerbated by the impairment of homeostatic mechanisms, by multimorbidity, and as a result older people are more prone to adverse drug reactions and, generally, these are more severe.^50^ Adverse drug reactions are a leading cause of hospitalisation, mortality, falls, fractures and hypoglycaemia, but symptoms are not always reported by patients as they are often mistaken for the symptoms of multiple diseases or with ageing. Prescribing the lowest effective doses of medication to elderly patients can help avoid adverse drug reactions, minimise side effects and thus encourage compliance.^51^ It is appropriate to remember the old aphorism ‘start low and go slow’.

Mr A is on a long-acting anticholinergic and we need to ask specifically about the most common adverse events: dry mouth, constipation, headache, pharyngitis and urinary retention. A slight increase in the risk for cardiovascular events has been found in the past with inhaled anticholinergic drugs^52^ and the risk/benefit balance needs to take into account his coronary heart disease. Recent reviews found a wide disparity in findings among the published studies evaluating the cardiovascular risks of inhaled anticholinergic agents.^53^ Tiotropium has no frequent inhibitory effect on cytochrome P450 isozymes and no clinically significant interactions have been reported.^54^ On the other hand, no studies have been found in the literature on the effect of age and severe renal or hepatic impairment on tiotropium pharmacokinetics.^54^

Notwithstanding the low oral bioavailability, inhaled β_2_-agonists have the potential to cause systemic adverse effects; therefore, we have to look for symptoms such as tremor, tachycardia, palpitations and changes in blood glucose or plasma potassium concentrations.^54^

## Minimising medication problems

A regular medication review by primary care physicians should be performed to reduce the risk for polypharmacy among those with multimorbidity.^55^ Nine key questions have been suggested^56^ that we should always ask our patients before a new prescription or at least once a year (Table 4):

1. Is each medication necessary?
2. Is the drug contraindicated in the elderly?
3. Are there duplicate medications?
4. Is the patient taking the lowest effective dosage?
5. Is the medication intended to treat the side effect of another medication?
6. Can the drug regimen be simplified?
7. Are there potential drug interactions?
8. Is the patient adherent?
9. Is the patient taking over-the-counter medication, a herbal product or another person’s medication?

Prescribing for multimorbid—and especially elderly—patients requires a careful and case-by-case assessment of the following specific elements: pharmacokinetics, pharmacodynamics, polypharmacy, comorbidity, adverse drug reactions, drug interactions, the delivery system, and the social and economic factors that affect nutrition and medication adherence.^56^ All of these factors can have an impact on outcomes.

The primary care physician has the challenging role of integrating all the clinical problems affecting the patient and reviewing all the medications taken by the patient at any point in time. This is the key to preventing polypharmacy and its consequences.

## Handling complex consultations on comorbidity

Mr A may present to the practice in one of three ways: (a) as an acute presentation of one of his symptoms—e.g., breathlessness; (b) with persistent chronic symptoms (in his case, cough, breathlessness and insomnia); or (c) for routine review. This commentary will focus on the latter two types of consultation.

## Consultation models:

The Stott and Davis model^57^ for the consultation prioritises four aspects of consultation:

- Dealing with the primary complaint (in this case scenario, symptoms of breathlessness, cough and insomnia).
- Management of the continuing problems (COPD and the multimorbidities).
- Using the consultation for opportunistic health promotion (e.g., giving up smoking and reducing alcohol consumption).
- Modification of health-seeking behaviour by encouraging supported self-management.

The consultation model of Pendleton *et al.*^58^ sets out seven ‘tasks’ for the consultation based around recognising the patient’s agenda—i.e., what they want out of the consultation, the effect of the problem (insomnia, breathlessness and cough) on the patient’s life and the production of shared management decisions.

In the United Kingdom, doctors receive payments under the Quality and Outcomes Framework, which rewards practices for ticking process boxes.^59^ For example, has the FEV_1_ or blood pressure been checked? As a consequence, chronic disease reviews tend to be driven by ‘ticking boxes’, risking neglect of the patient perspective.

## Issues faced by the doctor and patient in a consultation with multimorbidity

**Figure 1:**
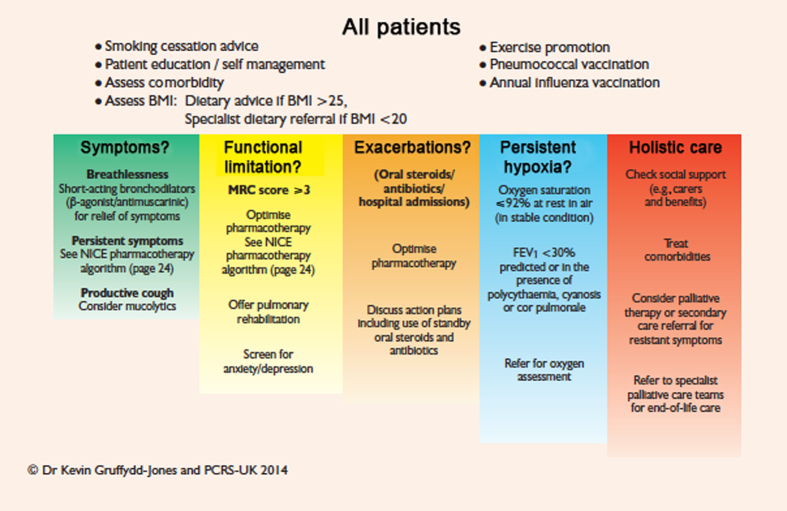
Patient-centred approach for the assessment of patients with COPD.

The PCRS-UK assessment algorithm for patients with COPD (Figure 1) taken from ‘The Diagnosis and Management of COPD in primary care’^60^ promotes a patient-centred approach to care, including a section on ‘holistic care’. This emphasises the importance of multimorbidities and psychosocial problems, including the needs of any caregivers.

**Figure 2:**
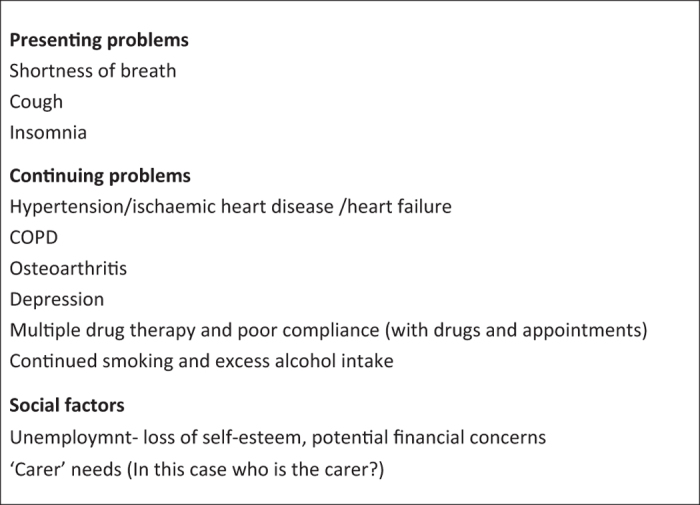
Issues for the consultation arising out of the case vignette.

From the information in the case vignette the issues appear to be as shown in Figure 2.

It is impossible to deal with these problems in the usual 10-min general practitioner consultation in the United Kingdom or even in a 20-min chronic disease management review by the practice nurse. Repeated attendance for multiple disease-specific chronic reviews is due to inefficient use of time by the primary care team and can be hugely inconvenient for the patient. So how might this practical problem be overcome?

## Managing multimorbidity in primary care

The following is derived from an excellent recent review article in the *BMJ*,^61^ ‘Managing multimorbidity’ in primary care, and also from a survey of UK practices that illustrates how practices are carrying out chronic disease reviews on patients with COPD and multimorbidities.^62^

A Cochrane review of interventions to improve outcomes in patients with multimorbidity in primary care illustrated the paucity of studies available.^63^ The limited evidence available shows that interventions are more successful if they deal with functional outcomes (‘how much more can you do’) rather than disease-specific outcomes. Also interventions that deal with common risk factors (in this case, vignette smoking and alcohol) can be successful.

The management of Mr A will depend on the timing of consultations and the organisation of care in different general practice surgeries. However, the general principles are likely to follow the Stott and Davies Model.

Investigation of the presenting problem: on the surface, this is likely to be cough and breathlessness, but the most pressing issue for the patient may be his tiredness, depression or insomnia.

Initial consultation: the history, examination and initiation of investigations (e.g., chest X-ray and full blood count) of the presenting complaint will be the main priority.

Ongoing problems: the medical and psychosocial comorbidities outlined in Figure 2 are likely to require an extended follow-up consultation with the general practitioner where issues such as polypharmacy can be addressed. It is important that Mr A see the same doctor to ensure continuity of care and maximise the success of any intervention.

## Health promotion/opportunistic screening

Once all the medical and psychosocial factors have been taken into consideration the barriers to dealing with Mr. A’s excess alcohol intake, smoking and non-attendance can be more easily addressed. It might be possible to draw up a shared management plan based on Mr. A’s views and priorities.

Effective management of the various problems is likely to involve referral to other members of the primary health-care team (e.g., social worker, psychological counsellor and smoking cessation services) and attendance for chronic disease management checks encouraged by combining the nurse-led reviews of his COPD, heart failure and hypertension.

## Organisational factors

The following have been used to improve the managements of patients with multimorbidity:^62,63^

- Increasing continuity of care by seeing the same doctor/practice nurse for review.
- Having specific extended consultation times (e.g., 30 min) for patients with complex problems.
- Use of postal, telephone and text reminders for appointments.
- Use of a common chronic disease management template that ideally can be shared with secondary and community care teams.
- Involvement of other members of the primary health are team—e.g., social worker, practice-attached psychologist, pharmacist and smoking cessation service.
- Involvement of specialist community teams in the multimorbidity clinic.
- Housebound or nursing home patients often have complex problems, and models of care include review by a named GP, district nurse or community matron.

## Shared decision-making

This involves the patient in management decisions within the consultation. A specific model using ‘Ariadne Principles’ has been devised for multimorbid consultations.^64^ This involves:

- Setting realistic targets (e.g., improving breathlessness so that the patient can walk to the car).
- Prioritising the problems that the patient has (‘Most of all I want to improve my insomnia’).
- Developing a management plan after discussing options with the patient.

A good opening question could be ‘What is bothering you most?’ or ‘What would you like to focus on today?’

A development in patient-centred care in the context of chronic disease management is that of care planning.^65^

This was originally used in the management of a single disease, diabetes, and involves the patient seeing the practice nurse for the required tests (e.g., blood pressure and glycosylated haemoglobin) in advance of an extended consultation with the GP. The patient is sent the results of the tests and then asked to prioritise their problems in anticipation of the doctor’s appointment.

This concept can be extended to the patient with multimorbidities using a unified multi-disease management template that can be tailored to the individual patient.

Such an approach has been used successfully for COPD patients, with multimorbidities in primary care, in Bradford in the north of England.^6^

**Figure 3:**
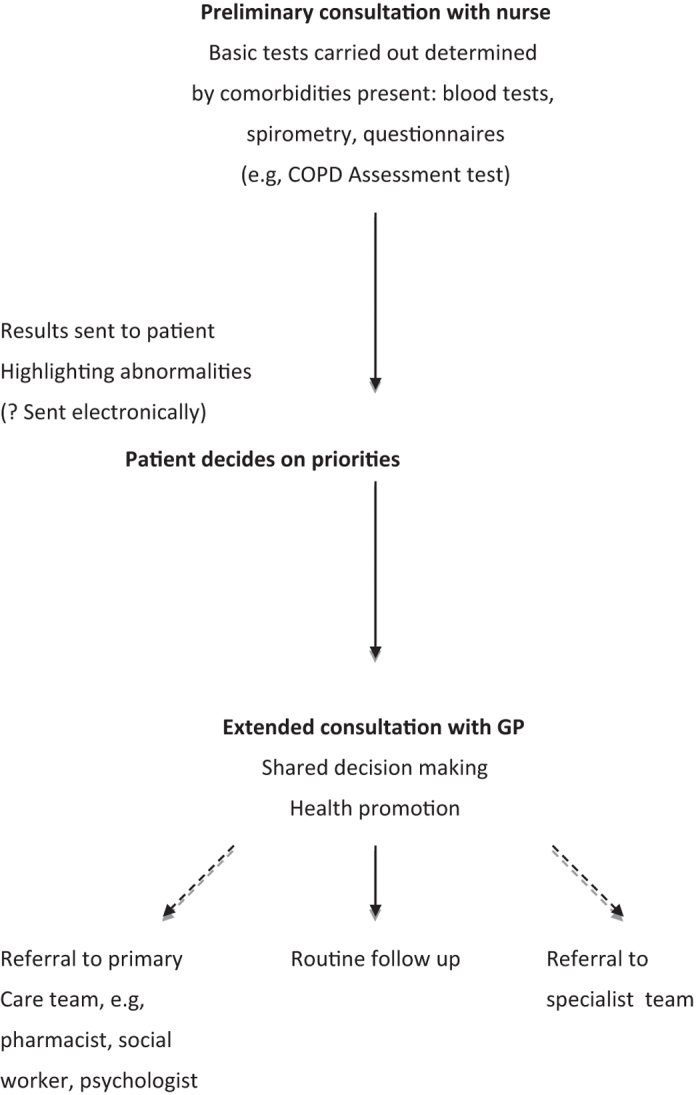
A model of care planning for patient with multimorbidity.

A model for structured chronic disease management of the patient with COPD and multiple comorbidities is shown in Figure 3. 

## Summary

- Management of the patient with multimorbidity involves consideration of the psychosocial as well as physical problems.
- A 10-min consultation, common in the United Kingdom, can deal with the patient’s presenting problem and can offer an opportunity for brief health promotion (e.g., giving up smoking), but an extended consultation time of at least 30 min, as in some European countries, is needed to deal with complex multiple conditions.
- Although chronic disease targets and tick boxes may aid structured reviews, considering the patient’s agenda including shared decision-making are key to producing worthwhile change.
- Multimorbid chronic disease management can be helped by the use of care planning, unified disease templates, use of information technology with appointment reminders, and help of the wider primary care and community teams.

## Figures and Tables

**Table 1:** Most common comorbidities in asthma and COPD

*Most common comorbidities in patients with asthma*		
Hypertension	17,000	16.2%
Depression	14,662	14.0%
Pain	13,480	12.8%
Bronchitis	12,030	11.5%
Dyspepsia	9,313	8.9%

*Most common comorbidities in patients with COPD*		
Hypertension	18,349	32.9%
Pain	12,720	22.8%
CHD	10,813	19.4%
Depression	9,997	17.9%
Dyspepsia	7,123	12.8%

Unpublished data from the data set reported in Barnett *et al.*^3^ based on a nationally representative sample of 1,751,841 primary care patients in Scotland, which had a total of 105,054 (6%) patients with asthma and 55,792 (3.2%) with COPD. The results above are for all patients with the index condition (asthma or COPD).

Abbreviations: CHD, coronary heart disease; COPD, chronic obstructive pulmonary disease.

**Table 2:** Inappropriate prescription in respiratory pharmacology: START and STOPP criteria

*STOPP*	*START*
Drug prescriptions potentially inappropriate in persons aged ⩾65 years	Medications to be considered for people aged ⩾65 years with the following conditions when no contraindications to prescription exist
1. Theophylline as monotherapy for COPD	1. Regular inhaled β_2_-agonist or anticholinergic agent for mild-to-moderate asthma or COPD
2. Systemic corticosteroids instead of inhaled corticosteroids for maintenance therapy in moderate-to-severe COPD	2. Regular inhaled corticosteroid for moderate-to-severe asthma or COPD when the predicted FEV_1_<50%
3. Nebulised ipratropium with glaucoma	3. Continuous oxygen at home with documented chronic type 1 respiratory failure or type 2 respiratory failure

Abbreviations: COPD, chronic obstructive pulmonary disease; STOPP, Screening Tool of Older Persons’ Prescriptions; START, Screening Tool to Alert doctors to the Right Treatment.

**Table 3:** The STOPP/START and Beers Criteria applied to Mr. A’s medication list

*Problem*	*Medication*	*STOPP/START criteria*		*Beers criteria*
		*STOPP*	*START*	*Recommendation*
Hypertension	Lisinopril	ACE inhibitors or angiotensin receptor blockers in patients with hyperkalaemia	ACE inhibitor with systolic heart failure and/or documented coronary artery disease.	
				
General osteoarthritis	Diclofenac	NSAID’s if eGFR <50 ml/min/1.73 m^2^ (risk of deterioration in renal function). Non-COX-2 selective NSAID with the history of peptic ulcer disease or gastrointestinal bleeding, unless with concurrent PPI or H2 antagonist (risk for peptic ulcer relapse). NSAID with established hypertension (risk for exacerbation of hypertension) or heart failure (risk for exacerbation of heart failure). Long-term use of NSAID (>3 months) for symptom relief of osteoarthritis pain where paracetamol has not been tried (simple analgesics preferable and usually as effective for pain relief)		Avoid chronic use unless other alternatives are not effective and the patient can take a gastro-protective agent (proton-pump inhibitor).
				
Coronary heart disease/heart failure	Aspirin	Aspirin with no history of coronary, cerebral or peripheral arterial occlusive symptoms. Long-term aspirin at doses greater than 160 mg per day (increased risk for bleeding, no evidence for increased efficacy) Aspirin with a past history of peptic ulcer disease without concomitant PPI (risk for recurrent peptic ulcer).		Aspirin for primary prevention of cardiac events. Lack of evidence of benefit versus risk in individuals ⩾80 years old. Use with caution in adults ⩾80 years old.
	Simvastatin		Statin therapy with a documented history of coronary, cerebral or peripheral vascular disease, unless the patient’s status is end of life or age is >85 years.	
	Bisoprolol	Beta-blocker with symptomatic bradycardia (<50/min), type II heart block or complete heart block (risk for profound hypotension, asystole).	Beta-blocker with ischaemic heart disease. Appropriate beta-blocker (bisoprolol, nebivolol and metoprolol orcarvedilol) with stable systolic heart failure.	
				
COPD	Tiotropium	Antimuscarinic bronchodilators (e.g., ipratropium and tiotropium) with a history of narrow angle glaucoma (may exacerbate glaucoma) or bladder outflow obstruction (may cause urinary retention).	Regular inhaled β_2_-agonist or antimuscarinic bronchodilator (e.g., ipratropium and tiotropium) for mild-to-moderate asthma or COPD.	These medications may cause aggravated prostate problems and make urination more difficult. Avoid in men with prostate problems.
	Salbutamol			
				
Depression/sleep problems	Sertraline	Selective serotonin re-uptake inhibitors with current or recent significant hyponatraemia, i.e., serum Na+<130 mmol/l (risk for exacerbating or precipitating hyponatraemia).		
	Zolpidem	Hypnotic Z-drugs (e.g., zopiclone and zolpidem; may cause protracted daytime sedation and ataxia).		Avoid chronic use (>90 days)

Abbreviations: ACE, angiotensin converting enzyme; COPD, chronic obstructive pulmonary disease; NSAID, non-steroidal anti-inflammatory drug; STOPP, Screening Tool of Older Persons’ Prescriptions; START, Screening Tool to Alert doctors to the Right Treatment.

**Table 4:** Mnemonics to reduce polypharmacy in the elderly (adapted from Skinner *et al.*)^56^

SAIL (1998)	S simple; prescribing drugs that can be taken once a day or adding a combination pill when a second pill must be added keeps a patient’s drug regimen uncomplicated. A adverse; the clinician must have knowledge of the adverse effects of all the drugs a patient is taking to avoid medication interactions I indication; there must be a clear indication for each drug a patient is taking with a desired therapeutic goal in mind L is for list; the patient’s medication list must be accurate, including OTC products, herbs, and alternative medications, and must correspond to their medical diagnoses.
ARMOR (2009)	A assess the individual for the total number of medications and for certain groups of medications that have potential for adverse outcomes in the older adult, such as beta-blockers, antipsychotics and antidepressants R review for possible drug–drug, drug–disease and drug–body interactions M minimise nonessential medications that lack a clear indication; the risks outweigh the benefits that could have a negative outcome on primary functions such as appetite, bladder/bowel, activity and mood O optimise by addressing duplication of drugs, adjustment of drugs for renal and hepatic function, reducing oral hypoglycaemia, and monitoring anticoagulants and seizure medications carefully R reassessment of the patient’s vital signs, cognitive status, function and medication compliance
TIDE (2012)	T time; allow sufficient time to address and discuss medication issues during each encounter I individualise; apply pharmacokinetic and pharmacodynamic principles to regimens by adjusting doses for renal and hepatic impairment and starting medications at the lowest effective dose D drug interactions; consider potential drug–drug and drug–disease interactions E educate; educate the patient and caregiver about non-pharmacological and pharmacologic treatments along with side effects and monitoring parameters
MASTER (2011)	M minimise drugs used A alternatives that should always be considered, especially non-drug therapies S start low and go slow T titrate therapy, adjusting dose based on individual response E educate the patient and family member with clear, written instructions R review regularly

**Table 5 tbl5:** 

***Mr A’s usual medication***	
***Problem***	Medication
Hypertension	Lisinopril 20 mg, 1 daily
General osteoarthritis	Diclofenac 75 mg as needed
Coronary heart disease/heart failure	Acetylsalicylic acid 75 mg, 1 daily, simvastatin 20 mg, 1 daily
	Bisoprolol 2.5 mg, 1 daily
COPD	Tiotropium 2.5 μg, 1 puff daily,
	Salbutamol 100 μg, 2 puffs as needed
Depression/sleep problems	Sertraline 50 mg, 1 daily
	Zolpidem 5 mg occasionally
Alcohol/smoking	No medication/brief advice

## Notes

S.M. received royalties from the sale of the book cited in this article, the ABC of Multimorbidity. The remaining authors declare no conflict of interest.
